# Systematic interventions based on the stress-induced situation, affective, bodily, and cognitive reactions framework to mitigate psychological distress in lung cancer patients post-thoracoscopic surgery: a randomized clinical trial

**DOI:** 10.3389/fpsyg.2025.1511622

**Published:** 2025-08-29

**Authors:** Lili Tang, Quan-Xing Liu, Yuexia He, Huanzhi Peng, Maoyu Luo, Hong Zheng, Qingling Zhang

**Affiliations:** ^1^Department of Thoracic Surgery, Second Affiliated Hospital of Army Medical University, Chongqing, China; ^2^Department of Medical Psychology, Second Affiliated Hospital of Army Medical University, Chongqing, China

**Keywords:** lung neoplasms, thoracoscopic surgery, psychological distress, quality of life, cognitive-behavioral therapy

## Abstract

**Objective:**

To evaluate the efficacy of a multi-component intervention based on the Situational, Affective, Bodily, and Cognitive (SABC) stress-response model in reducing psychological distress and enhancing quality of life among post-thoracoscopic lung-cancer patients.

**Methods:**

In this single-center, assessor-masked, parallel-group randomized controlled trial, 240 patients were randomized (1:1) to either a 12-month SABC intervention (*n* = 120) or standard care (*n* = 120) by means of computer-generated, sealed-envelope allocation. Outcome assessors were blinded to group assignment. The intervention comprised psycho-education, SABC-based skills training, a daily-habits checklist and scheduled follow-up. Psychological distress was measured with the NCCN Distress Thermometer (DT); quality of life was assessed with the EORTC QLQ-C30 at baseline and at 1, 3, 6, and 12 months post-surgery. Linear mixed-effects models were used, under both intention-to-treat (ITT) and per-protocol analyses, to test time-by-group interactions.

**Results:**

Time-by-group interactions favored the intervention group for physical (ITT: *F* = 5.632, *P* < 0.001; PP: *F* = 6.084, *P* < 0.001), role (ITT: *F* = 3.325, *P* = 0.010; PP: *F* = 2.675, *P* = 0.031) and emotional functioning (ITT: *F* = 5.543, *P* < 0.001; PP: *F* = 5.566, *P* < 0.001), and for the distress thermometer (ITT: *F* = 3.791, *P* = 0.005; PP: *F* = 5.258, *P* < 0.001). Social functioning improved in the ITT analysis only (*P* = 0.016); fatigue improved in the per-protocol analysis only (*P* = 0.008). No significant differences were observed for cognitive functioning, nausea and vomiting, pain, dyspnoea, insomnia, appetite loss, constipation, diarrhea, financial difficulties or global health status (*P* > 0.05).

**Conclusion:**

A structured SABC-informed intervention significantly improves functional domains and reduces psychological distress in early-stage lung-cancer survivors after thoracoscopic surgery. Limitations include recruitment from a single tertiary center and exclusion of patients with severe comorbidities, which may limit generalisability to broader clinical populations.

**Clinical Trial Registration:**

A randomized controlled trial of a psychological-distress intervention in patients with lung cancer based on the SABC framework. https://www.chictr.org.cn/searchproj.html?title=&officialname=&subjectid=&regstatus=&regno=ChiCTR1900028487&secondaryid=&applier=&studyleader=&createyear=&sponsor=&secsponsor=&sourceofspends=&studyailment=&studyailmentcode=&studytype=&studystage=&studydesign=&recruitmentstatus=&gender=&agreetosign=&measure=&country=&province=&city=&institution=&institutionlevel=&intercode=&ethicalcommitteesanction=&whetherpublic=&minstudyexecutetime=&maxstudyexecutetime=&btngo=btn, identifier: ChiCTR1900028487.

## 1 Introduction

In China, lung cancer remains the leading cause of cancer-related morbidity and mortality. Following diagnosis, patients frequently experience clinically significant psychological distress (Han et al., 2024), manifesting as heightened anxiety, depressive symptoms and diminished quality of life. Histopathologically, lung cancer is classified as non-small cell lung cancer (NSCLC) or small cell lung cancer (SCLC), with NSCLC comprising ~85% of cases (Ruiz-Cordero and Devine, 2020; Chen et al., 2022). Surgical resection is the most effective curative treatment for early-stage disease; however, surgical trauma and postoperative complications often result in psychological distress and physical symptoms such as pain, cough, dyspnoea and fatigue, which substantially impair patients' quality of life and constitute a major public health challenge (Wu et al., 2021).

Psychological distress can be caused by a variety of factors, including psychological, social, spiritual and physical factors. Various national and international organizations have endorsed distress as the sixth vital sign, including the International Psycho-Oncology Society, the Union for International Cancer Control, the Canadian Association of Psychosocial Oncology and the Canadian Association of Nurses in Oncology (Fitch et al., 2018). Chronic stress induces physiological distress by altering the neuroendocrine and sympathetic nervous system signaling pathways, thereby disrupting homeostasis (Cui et al., 2021). In the context of cancer, psychosocial distress exacerbates inflammation and oxidative/nitrosative stress, diminishes immune surveillance, and induces dysfunction of the autonomic nervous system and the hypothalamic-pituitary-adrenal axis (Bortolato et al., 2017). The confluence of these factors, coupled with the experience of psychological distress, accelerates tumor growth and metastasis, consequently reducing patient survival rates (Grassi et al., 2013).

Studies indicate that 11.2% of still report that they experience psychological distress 20 years or more after a cancer diagnosis (Abdelhadi et al., 2022). The prevalence of psychological distress among patients with differentiated thyroid carcinoma (DTC) ranges from 13.8 to 29.33% (Husson et al., 2020; Gao et al., 2022), breast cancer patients from 46 to 50% (Tang et al., 2024; Adeyemi et al., 2021), and lung cancer patients from 41.9 to 63.75%. Notably, lung cancer patients exhibit a relatively higher rate of psychological distress.

Emerging evidence indicates that multi-component prehabilitation—initiated during the perioperative window—can simultaneously enhance physical performance and ameliorate anxiety and depression in cancer populations. A systematic review by Gennuso et al. (2024) synthesized data from multiple randomized trials and demonstrated that structured prehabilitation programmes, incorporating exercise, psychoeducation and nutritional support, produce clinically meaningful reductions in both anxiety and depressive symptoms, alongside improved functional capacity. These findings underscore the broader relevance of introducing systematic, theory-driven interventions around the time of surgery to optimize post-operative biopsychosocial outcomes.

Interventions targeting psychological distress in patients with lung cancer have predominantly centered on psycho-education, mindfulness training, and aerobic-exercise programmes (Tian et al., 2022; Takemura et al., 2024; Ramos Diaz et al., 2014; Horn et al., 2025). For example, nurse-led psychological interventions have demonstrated non-significant efficacy among breast-cancer patients experiencing moderate-to-severe distress (Bidstrup et al., 2023); Mindfulness-based courses customarily extend over 8 weeks, with individual sessions lasting 3 h; sustained engagement is impeded by the protracted format, resulting in suboptimal adherence (Ojeda Valencia et al., 2023; Zaki et al., 2022; Baltruschat et al., 2021; Girelli et al., 2024). Patients with lung carcinoma frequently experience fluctuating symptom burden, restricted prognostic trajectories and treatment-associated stressors. An intervention is therefore required that is temporally economical yet capable of rectifying maladaptive cognitions and concomitantly enhancing physiological functioning. The SABC framework, grounded in chronic-illness self-management theory and transactional stress-and-coping paradigms, sequentially targets four mechanistic domains: (1) active stressor engagement; (2) attenuation of amygdala hyper-reactivity; (3) modification of cognitive appraisal; and (4) augmentation of physical activation. Cognitive-behavioral techniques are embedded throughout these phases to elicit enduring cognitive and behavioral modifications that remodel aberrant neural circuitry, thereby attenuating affective distress and somatic symptomatology, correcting cognitive distortions and refining behavioral repertoires to reduce psychological morbidity.

Given the relative paucity of research applying the SABC model to psycho-oncology in lung cancer, the present study analyzed the framework across four empirically derived dimensions-contextual regulation, emotional regulation, somatic activation and cognitive restructuring-to examine its effects on post-operative amygdala responsivity, cognitive bias and physical symptomatology. Findings are intended to furnish empirical evidence for refining psychophysiological integration within post-surgical recovery pathways for this population.

## 2 Methods

This randomized controlled trial took place in the Thoracic Surgery Department of a Chinese tertiary hospital. Lung cancer patients newly admitted to the study were enrolled to complete baseline assessments using the psychological distress thermometer (DT) and European Quality of Life Questionnaire C30 (QLQ-C30) within 2 days post-admission (Time Point 1). These evaluations were conducted by trained bedside nurses, who also gathered demographic and medical data. Eligible patients were then randomized into either a control or an intervention group by senior nurses with specific training for this study. Each nurse attended a structured eight-session training course (one 2-h session per week for eight consecutive weeks) devised and delivered by consultant liaison-psychiatrists and senior clinical health psychologists. The syllabus comprised (i) theoretical instruction on stress–cognition–behavior pathways, and (ii) systematic rehearsal of each SABC component to ensure fidelity and safe practice. The allocation sequence was computer-generated (permuted blocks of 10, 1:1 ratio) and the list was sealed in sequentially numbered, opaque envelopes by an independent statistician who had no involvement in patient recruitment, baseline assessment, or clinical care. Envelopes were opened only by senior nurses after each participant had provided written informed consent. Group assignment was revealed by envelope number: odd numbers indicated standard care (control), and even numbers indicated comprehensive care (intervention). Follow-up assessments were conducted at 1 month (Time Point 2), 3 months (Time Point 3), 6 months (Time Point 4), and 12 months (Time Point 5) post-intervention initiation.

### 2.1 Inclusion and exclusion criteria

The inclusion criteria were: (1) age ≥18 years; (2) first admission for lung cancer with malignant lung nodules; (3) type of surgery: thoracoscopic minimally invasive surgery; (4) expected survival > 1 year; (5) being able to read, talk, and write Chinese; (6) consent to participate in this study; (7) Hospital Anxiety and Depression Scale (HADS) total score ≥15, or Hospital Anxiety Scale anxiety score ≥9, or Hospital Depression Scale depression score ≥9. The exclusion criteria were: (1) hearing impairments or communication disorders that prevent completion of the questionnaire; (2) having received psychological counseling and psychological treatment within the past 3 months; (3) currently taking anti-anxiety medication or having taken an antidepressant in the last 3 months.

### 2.2 Sample size

The required sample size was initially calculated using established formulae and estimates derived from the psychological-intervention literature in cancer populations, indicating 25 participants per group (Mertz et al., 2017).


$$\begin{array}{l} n_{1}=n_{1}=2{\left[\frac{\left(z_{\alpha }+z_{\beta }\right)\sigma }{\delta }\right]}^{2},\sigma^{2}=\frac{\left(s_{1}^{2}+s_{2}^{2}\right)}{2}\alpha =0.05,\;\beta =0.1 \end{array}$$

Accounting for the marked heterogeneity of cancer patients (Shin et al., 2017), the protracted intervention period and anticipated attrition during follow-up, together with recommendations that a minimum of 200 participants is required for robust statistical inference, we enrolled 240 participants and randomly allocated them in a 1:1 ratio to the intervention or control group.

### 2.3 Intervention program

#### 2.3.1 Psychological education

Prior to discharge, senior nurses delivered a 40-min mental-health education session on stress mechanisms to patients and their families. The session addressed: (1) stress-coping theory; (2) the adverse emotional, physical and cognitive effects of persistent stress; and (3) key lifestyle modifications—reducing caffeine intake, ensuring adequate sleep, developing a stress-resilient mindset and adopting a stress-resistant diet. Patients received audio recordings of guided meditation and body-scan exercises and a self-help manual on the prevention of anxiety and depression. To enhance adherence, senior nurses added patients on WeChat for follow-up reminders and support.

#### 2.3.2 SABC model system intervention measures

Telephone follow-up: trained nurses call up to 20 patients per day (3–5 min each) and conduct a weekly group assessment of symptoms, intervention adherence and deliberate practice. If a call is unanswered, a WeChat SMS reminder is sent. For details of the SABC model system intervention measures, please see Table 1.

**Table 1:** SABC intervention components.

**Component (S-A-B-C)**	**Delivery personnel**	**Frequency**
S–Stress Behavioral Responses	Research family members	family members
A–Amygdala-Dominated Affective Responses	Participant (self-guided), audio script provided by research team	10–15 min, once-daily schedule
B–Bodily Sensation Responses	Participant,Walking/Yoga/Tai Chi,	≥30 min/session, ≥5 days/week
C–Cognitive Responses	Participant (self-guided), audio script provided by research team	5–10 min, twice daily
Daily Habit Reinforcement	Automated WeChat group assistant + nurses	Continuous

#### 2.3.3 Control program

The control group received standard postoperative care, consisting of:

- postoperative health education covering cough management, pain control, functional exercise (video-guided) and dietary guidance; and
- a single follow-up telephone call from a nurse seven days after discharge.

On study completion, participants were given complimentary resources: audio recordings of meditation and mindful body-scan exercises, and a handbook on anxiety- and depression-prevention.

### 2.4 Data collection and instruments

This study was prospectively registered with the Chinese Clinical Trial Registry (registration date: 22 December 2019; identifier: ChiCTR1900028487) and approved by the hospital's Research Ethics Committee (approval number: 2019-S-017).

Prior to enrolment, participants received a comprehensive study information sheet detailing the aims, procedures, and potential risks. Written informed consent was obtained from all participants. Data confidentiality was strictly maintained by trained clinical research nurses, with anonymised records stored in a secure, access-controlled database.

#### 2.4.1 Basic demographic data

Following a comprehensive literature review and expert consultation, we developed a standardized questionnaire to record participants' baseline characteristics. Data comprised: demographics (age, sex, education level, marital status and occupation); clinical parameters (TNM stage, 7th edition 2009 classification, and histopathological subtype). Stage and subtype were extracted from each patient's medical record. Baseline characteristics were comparable between groups (all *P* > 0.05). Demographic details have been reported by Zhang et al. (2023).

#### 2.4.2 DT and QLQ-C30

Psychological distress was measured using the National Comprehensive Cancer Network (NCCN) Distress Thermometer (DT)—a validated screening tool (Graham-Wisener et al., 2021; McFarland et al., 2020). The scale was translated into Chinese by Zhang Enning in 2010 and has since been validated among Chinese cancer patients; the total Cronbach's α coefficient is 0.948. Psychological distress was scored on a scale from 0 (no distress) to 10 (extreme distress) (Tan et al., 2019).

The European Organization for Research and Treatment of Cancer Quality of Life Questionnaire Core 30 (EORTC QLQ-C30, version 3.0) is a validated instrument for assessing cancer-specific quality of life. The standard Chinese version of the scale demonstrated good overall internal consistency; Cronbach's α exceeded 0.70 for all domains except cognitive functioning (α = 0.49) (Koch et al., 2021). It comprises 30 items distributed across 15 domains: five functional scales: physical, role, cognitive, emotional, and social functioning; three symptom scales: fatigue, pain, and nausea/vomiting; one global health status scale; six single-item symptom measures. Scoring: items 29 and 30 use a 7-point Likert scale (1 = “not at all ”to 7 =“very much”). The remaining 28 items use a 4-point Likert scale (1 = “not at all” to 4 = “very much”). Interpretation: higher scores on functional and global health scales indicate better quality of life. Higher scores on symptom scales/single items indicate greater symptom burden and poorer quality of life. The EORTC QLQ-C30 has demonstrated robust reliability and validity in evaluating postoperative quality of life among lung cancer patients across diverse age and sex groups.

### 2.5 Statistical analysis

Statistical analyses were performed using SPSS version 22.0 and RStudio version 1.2.5001 (R environment version 4.0.3; R Core Team, 2020). Baseline characteristics were compared between the intervention and control groups with independent-samples *t*-tests for continuous variables (age and body-mass index) and χ^2^ tests for categorical variables (sex, education level, family per-capita monthly income, marital status, TNM stage and tumor type), following the approach of a previous intervention study (Henrich et al., 2020), Drawing on the per-protocol cohort, we examined the longitudinal trajectories of DT and QLQ-C30 global scores across five time-points for participants in both treatment arms. We subsequently conducted an intention-to-treat analysis on the full dataset, using multiple imputation to handle missing data (Graham, 2009). The intervention effect was analyzed by fitting a linear mixed-effects model with age, sex, body-mass index and education level as covariates. Missing data were assumed to be missing at random (MAR). Multiple imputation by chained equations (MICE) was implemented in R using the “mice” package, producing 20 imputed datasets that were pooled according to Rubin's rules. All randomized participants were retained in the intention-to-treat (ITT) analysis according to their original allocation.

## 3 Results

### 3.1 Patient demographic and clinical characteristics

**Figure 1:**
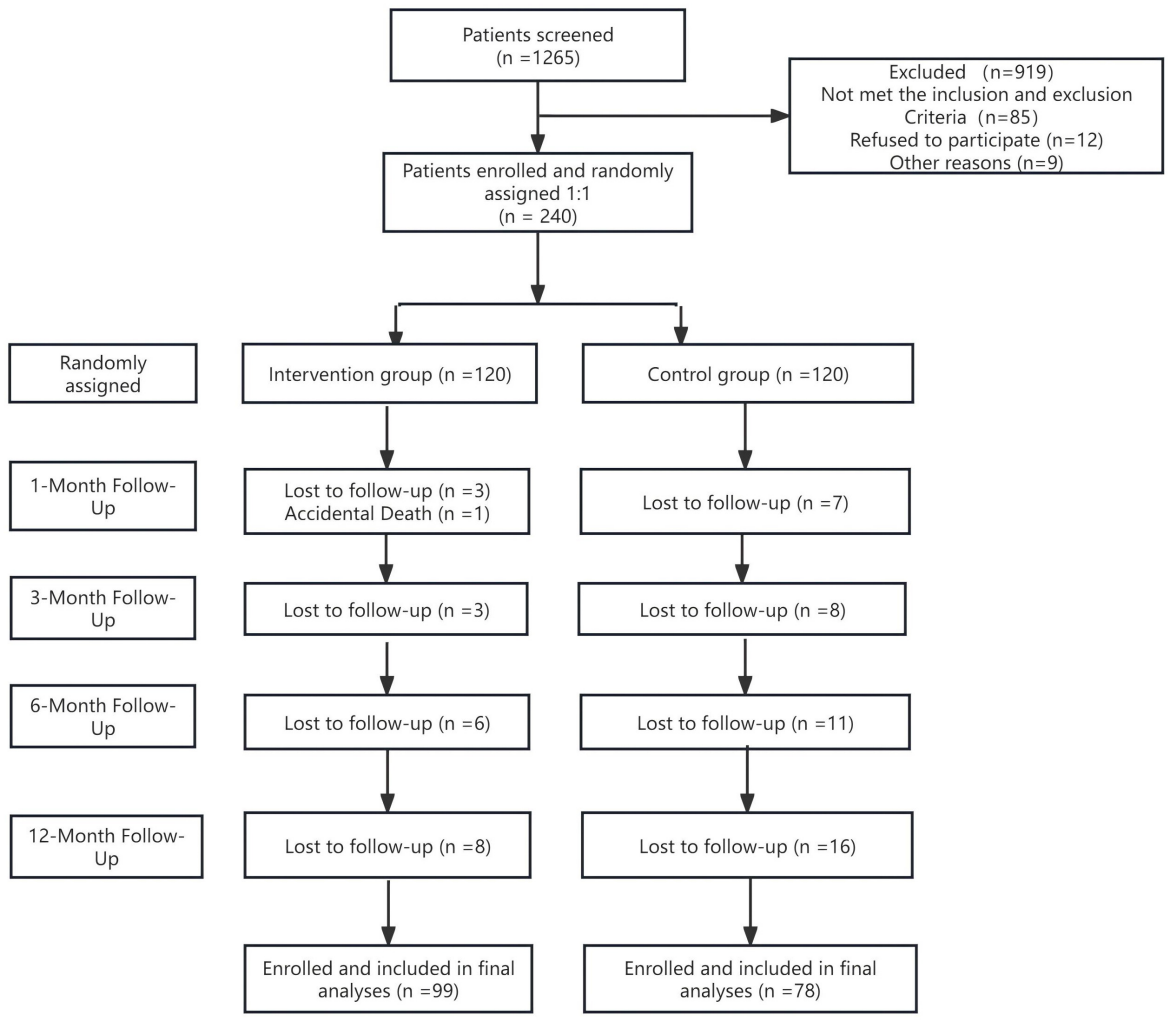
SABC study flow chart of participants assigned to intervention and control groups.

Baseline data were collected from January to December 2020, with post-intervention follow-up completed by December 2021. Of 240 participants, 13 confirmed lung-cancer cases were referred from respiratory and oncology departments; the remainder were recruited from outpatient clinics (Table 2). During the study period, one patient (0.83 %) in the intervention group died, and 20 (16.66 %) in the intervention group vs. 42 (35 %) in the control group withdrew. The intervention-group dropout rate was significantly lower (*P* = 0.002). Reasons for withdrawal included perceived burden of follow-up procedures, insufficient nurse-patient rapport and other medical issues. Intervention fidelity was maintained through weekly telephone follow-ups and daily SMS reminders delivered by WeChat group assistants. Approximately 86 participants received daily family hugs or expressions of gratitude, while 73 completed mindful body-scans. Overall, 26.25% (*n* = 63) withdrew from baseline to follow-up (Figure 1).

**Table 2:** Demographic and clinical characteristics of participants (*n* = 240).

**Variables**	**No (%) or x̄(s)**			***P*-value**
	**Intervention group (*****N*** = **120)**	**Control group (*****N*** = **120)**	**Overall (*****N*** = **240)**	
**Gender**				
Female	87.0 (72.5%)	79.0 (65.8%)	166 (69.2%)	0.535
Male	33.0 (27.5%)	41.0 (34.2%)	74.0 (30.8%)	
**Age (years)**				
≤ 30	10.0 (8.3%)	3.00 (2.5%)	13.0 (5.4%)	0.707
31–40	32.0 (26.7%)	32.0 (26.7%)	64.0 (26.7%)	
41–50	38.0 (31.7%)	34.0 (28.3%)	72.0 (30.0%)	
51–60	30.0 (25.0%)	40.0 (33.3%)	70.0 (29.2%)	
≥61	10.0 (8.3%)	11.0 (9.2%)	21.0 (8.8%)	
**Occupation**				
Clerk or civil servant	67.0 (55.8%)	57.0 (47.5%)	124 (51.7%)	0.871
Worker	43.0 (35.8%)	50.0 (41.7%)	93.0 (38.8%)	
Other	10.00 (8.3%)	13.0 (10.8%)	23.0(9.6%)	
**Spouse's occupation**				
Clerk or civil servant	68.0 (56.6%)	50.0 (41.7%)	118 (52.2%)	0.139
Worker	34.0 (28.3%)	54.0 (45.0%)	88.0 (36.7%)	
Other	18.0 (15%)	16.0 (13.4%)	34.0 (14.2%)	
**Marital status**				
Married	110 (91.7%)	114 (95.0%)	224 (93.3%)	0.667
Divorced	2.00 (1.7%)	2.00 (1.7%)	4.00 (1.7%)	
Widowed	4.00 (3.3%)	4.00 (3.3%)	8.00 (3.3%)	
Single	4.00 (3.3%)	0 (0%)	4.00 (1.7%)	
**Family relationship**				
Good	103 (85.8%)	103 (85.8%)	206 (85.9%)	0.792
Normal	15.0 (12.5%)	17.0 (14.2%)	32.0 (13.3%)	
Not good	2.00 (1.7%)	0 (0%)	2.00 (0.8%)	
**Husband-wife relationship**				
Good	109 (90.8%)	107 (89.2%)	216 (90.1%)	0.171
Normal	3.00 (2.5%)	10.0 (8.3%)	13.0 (5.4%)	
Not good	8.00 (6.7%)	3.00 (2.5%)	11.0 (4.6%)	
**Relationship of children**				
Good	107 (89.1%)	117 (97.5%)	224 (93.3%)	0.0448
Normal	6.00 (5.0%)	3.00 (2.5%)	9.00 (3.8%)	
Not good	7.00 (5.8%)	0 (0%)	7.00 (2.9%)	
**Knowledge of the disease**				
Yes	112 (93.3%)	115 (95.8%)	227 (94.6%)	0.694
No	8.00 (6.7%)	5.00 (4.2%)	13.0 (5.4%)	
**Time for sports activities (week)**				
Mean (SD)	0.442 (1.14)	0.433 (1.21)	0.438 (1.17)	0.998
Exceptional	11.0 (9.2%)	13.0 (10.8%)	24.0 (10.0%)	0.67
Good	41.0 (34.2%)	28.0 (23.3%)	69.0 (28.8%)	
Normal	24.0 (20.0%)	29.0 (24.2%)	53.0 (22.1%)	
Bad	40.0 (33.3%)	49.0 (40.8%)	89.0 (37.1%)	
Very bad	4.00 (3.3%)	1.00 (0.8%)	5.00 (2.1%)	

### 3.2 Effects of SABC intervention on DT and health related QoL of lung cancer patients post-thoracoscopic surgery

**Figure 2:**
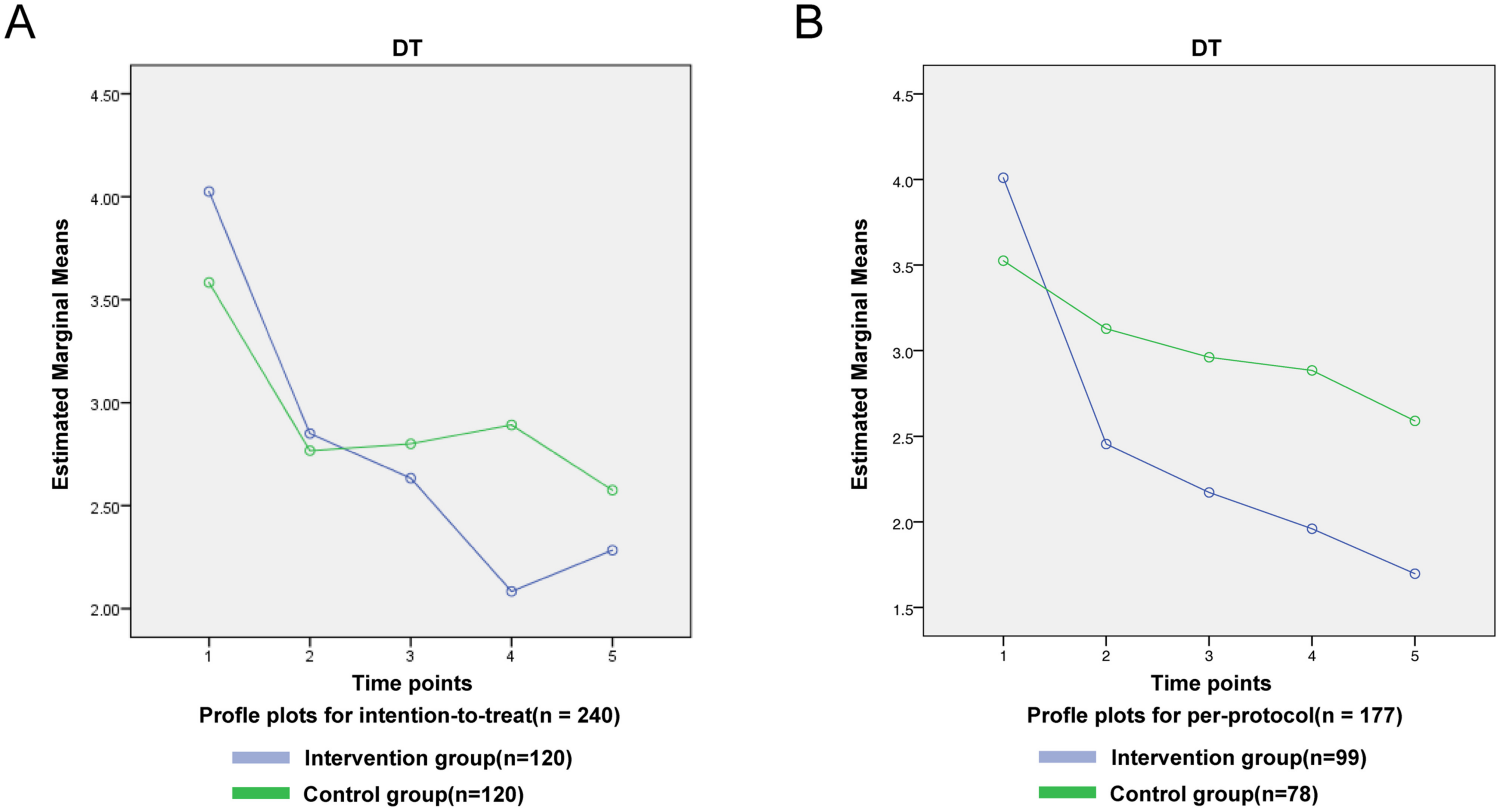
DT intent-to-treat outcomes line chart.

Outcomes were analyzed under both intention-to-treat (ITT; *n* = 240) and per-protocol (PP; *n* = 177) frameworks. Baseline characteristics were balanced between arms in both datasets (all *P* > 0.05; Table 3). The intention-to-treat (ITT) analysis showed a significant overall time-by-group interaction for DT (*F* = 3.791, *P* < 0.01), with the intervention group achieving lower DT scores than controls at 6 months (*t* = 3.715, *P* < 0.001) and 12 months (*t* = 2.180, *P* < 0.05); these effects were corroborated and strengthened in the per-protocol analysis (*F* = 5.258, *P* < 0.001), which revealed even larger intervention benefits at 6 months (*t* = 5.956, *P* < 0.001) and 12 months (*t* = 7.775, *P* < 0.001) (Figure 2).

**Table 3:** Mean scores for DT and QLQ-C30 at different time points and the results for per-protocol and intention-to-treat analyses.

**Outcome and group**	**T1**		**T2**		**T3**		**T4**		**T5**		**Overall**	**T1–T2**	**T1–T3**	**T1–T4**	**T1–T5**
	***M***	**SD**	***M***	**SD**	***M***	**SD**	***M***	**SD**	***M***	**SD**	***F***	***t***	***t***	***t***	***t***
**Intention-to-treat (IG** = **120, CG** = **120)**															
**DT**															
Intervention group	4.03	2.31	2.85	2.08	2.63	1.75	2.08	1.79	2.28	1.41	3.791^**^	1.065	1.808	3.715^***^	2.180^*^
Control group	3.58	2.45	2.77	1.99	2.80	1.90	2.89	2.06	2.58	1.77					
**QLQ-C30 (Functional domain)**															
**Physical function**															
Intervention group	83.28	11.85	78.61	15.09	82.83	13.35	84.89	13.82	87.61	18.05	5.632^***^	−1.019	−0.921	−1.490	−4.411^***^
Control group	85.06	11.40	77.28	16.64	82.00	15.50	81.33	16.34	80.33	23.28					
**Role function**															
Intervention group	88.47	17.53	83.19	18.77	88.06	17.71	87.78	18.55	91.94	13.66	3.325^*^	−2.583^**^	−2.912^**^	−2.489^*^	−3.240^**^
Control group	92.36	14.95	79.44	21.79	83.33	21.82	84.30	22.28	86.25	21.24					
**Emotional function**															
Intervention group	65.14	16.31	74.58	15.20	75.76	16.42	76.46	16.72	77.92	16.30	5.543^***^	−2.012^*^	−3.658^***^	−3.353^***^	−4.116^***^
Control group	67.01	16.18	71.88	15.28	69.31	15.09	70.70	15.80	70.42	16.66					
**Cognitive function**															
Intervention group	73.89	19.41	81.47	17.37	82.43	15.36	82.30	17.95	84.34	16.64	0.419	−0.083	−0.846	0.044	−0.843
Control group	71.81	19.78	79.17	14.44	78.14	13.19	80.33	13.19	80.06	14.32					
**Social function**															
Intervention group	78.75	20.26	79.31	19.68	81.11	17.41	84.17	18.31	86.25	15.97	3.080^*^	−2.772^**^	−2.533^*^	−3.059^**^	−2.581^*^
Control group	81.25	19.15	73.75	20.34	76.25	19.52	77.78	20.08	81.25	23.02					
**QLQ-C30 (symptom domain)**															
**Fatigue**															
Intervention group	31.13	23.43	32.06	20.11	30.04	18.97	29.86	21.89	26.50	20.67	1.286	0.575	1.492	1.051	2.074^*^
Control group	38.29	29.00	41.09	23.37	42.06	26.13	40.44	26.36	40.42	25.90					
**Nausea and vomiting**															
Intervention group	12.59	18.10	8.75	16.47	8.33	15.76	7.73	15.28	6.90	11.23	2.081	2.451^*^	1.997^*^	2.253^*^	1.047
Control group	12.87	18.16	14.77	18.70	13.29	17.47	13.29	17.48	9.63	14.33					
Intervention group	12.64	18.59	20.56	20.24	15.69	16.35	15.28	16.96	13.33	15.97	1.208	1.354	1.955	0.702	1.604
Control group	12.36	15.05	24.03	23.94	20.83	22.38	16.94	16.59	17.50	16.00					
**Dyspnea**															
Intervention group	15.55	18.80	27.92	21.95	20.83	18.11	21.53	19.73	15.14	21.50	0.987	1.350	1.698	0.348	1.045
Control group	16.67	16.17	33.33	25.47	27.36	23.25	23.75	19.16	19.58	17.77					
**Insomnia**															
Intervention group	29.44	26.70	32.78	26.28	28.61	24.94	27.78	26.40	25.55	25.10	2.036	2.186^*^	2.539^*^	1.622	0.987
Control group	27.50	24.32	39.44	33.75	36.67	30.98	32.22	26.26	27.50	23.54					
**Appetite loss**															
Intervention group	18.89	22.76	13.61	19.56	14.17	19.16	12.50	22.48	9.72	15.21	2.131	2.697^**^	1.131	2.088^*^	1.131
Control group	20.28	23.39	23.61	25.71	19.17	23.93	20.55	24.13	14.72	19.71					
**Constipation**															
Intervention group	78.75	20.26	79.31	19.68	81.11	17.41	84.17	18.31	86.25	15.97	0.063	0.389	0.389	0.097	0.292
Control group	81.25	19.15	73.75	20.34	76.25	19.52	77.78	20.08	81.25	23.02					
**Diarrhea**															
Intervention group	12.22	18.80	10.55	16.16	11.67	18.15	13.89	20.08	12.50	19.83	1.567	−1.211	−1.211	−2.421^*^	−1.715
Control group	16.39	19.80	11.39	16.45	12.50	17.32	11.39	17.01	11.94	16.62					
**Financial problems**															
Intervention group	26.67	22.70	24.44	26.55	20.28	21.73	20.83	22.48	17.22	20.72	0.833	0.868	1.542	0.386	1.350
Control group	25.83	29.14	26.11	28.40	23.89	27.39	21.11	25.17	20.28	23.79					
**Global health**															
Intervention group	64.03	25.61	63.54	20.95	66.25	22.91	67.01	23.43	67.78	23.74	0.630	−0.133	−0.550	−1.199	−1.166
Control group	56.32	32.25	55.28	26.94	56.25	30.22	54.31	29.05	55.21	34.04					
**Per protocol (IG** = **99, CG** = **78)**															
**DT**															
Intervention group	4.01	2.48	2.46	2.20	2.17	1.75	1.96	1.86	1.70	1.48	5.258^***^	4.207^***^	5.612^***^	5.956^***^	7.775^***^
Control group	3.53	2.16	3.13	2.10	2.96	1.96	2.89	1.89	2.59	1.73					
**QLQ-C30 (functional domain)**															
**Physical function**															
Intervention group	83.43	12.39	78.45	14.59	83.16	13.34	84.78	13.70	96.97	26.97	6.084^***^	5.107^***^	1.049	0.949	−2.572^*^
Control group	85.47	11.25	76.84	17.97	83.42	14.56	81.62	18.15	83.76	30.27					
**Role function**															
Intervention group	88.55	17.92	83.00	18.59	88.38	17.41	89.39	16.91	92.09	13.75	2.675^*^	4.265^***^	2.458^*^	1.953	0.723
Control group	90.60	16.90	78.42	22.49	81.62	23.35	82.26	24.37	84.40	23.78					
**Emotional function**															
Intervention group	65.15	17.31	74.58	15.85	76.52	15.31	76.77	16.20	78.62	16.24	5.566^***^	–−4.976^***^	–−4.847^***^	−4.978^***^	−5.093^***^
Control group	66.13	16.90	70.73	15.59	69.13	15.14	69.98	16.79	68.70	16.35					
**Cognitive function**															
Intervention group	73.06	20.30	81.31	18.48	82.32	16.12	83.00	18.13	84.34	17.30	0.167	−4.911^***^	–−5.665^***^	–−6.322^***^	−6.166–^***^
Control group	68.16	20.49	77.78	15.11	79.06	12.72	80.13	13.17	79.70	13.60					
**Social function**															
Intervention group	79.97	19.19	78.45	20.52	81.48	17.31	83.00	18.44	86.20	16.33	1.042	2.027^**^	0.663	−0.740	−1.713^*^
Control group	79.70	20.57	73.08	21.69	75.64	19.86	79.49	20.79	80.34	24.73					
**QLQ-C30 (symptom domain)**															
**Fatigue**															
Intervention group	24.69	19.08	28.62	15.64	26.04	14.82	25.14	15.60	23.12	16.16	3.464^**^	−4.365^***^	−2.769^**^	−2.209^*^	−1.796
Control group	22.79	17.99	34.90	20.08	31.62	21.79	30.62	21.13	31.48	21.37					
**Nausea and vomiting**															
Intervention group	8.42	16.04	6.23	14.20	7.24	15.09	6.40	14.43	5.39	10.05	0.573	0.526	0.545	0.841	1.691
Control group	7.27	15.80	8.12	13.89	7.05	12.75	7.27	10.94	6.20	11.11					
**Pain**															
Intervention group	13.47	19.59	22.05	19.02	16.50	16.58	15.32	16.27	13.64	15.31	1.269	−5.473^***^	−3.548^***^	−1.975	−1.319
Control group	15.39	15.61	29.27	25.79	24.57	21.61	19.66	14.89	19.02	12.80					
**Dyspnea**															
Intervention group	14.48	19.14	28.62	21.83	21.21	18.10	20.54	19.46	12.12	18.10	1.277	−7.289^***^	−4.401^***^	−3.362^**^	−0.655
Control group	17.52	17.59	35.04	27.34	26.07	23.81	23.50	20.18	22.22	19.12					
**Insomnia**															
Intervention group	31.31	28.10	34.34	27.54	28.62	23.33	27.94	23.67	25.59	23.73	1.470	−2.761^**^	−0.881	0.183	1.241
Control group	32.05	26.56	44.44	36.70	38.89	32.42	34.61	28.65	32.48	25.18					
**Appetite loss**															
Intervention group	15.82	21.48	13.47	19.59	12.79	17.63	11.45	21.38	9.76	15.25	1.274	−0.081	1.006	1.546	2.359^*^
Control group	14.53	19.06	18.80	20.52	14.10	18.23	13.25	18.09	12.82	18.00					
**Constipation**															
Intervention group	14.68	21.00	16.51	20.60	15.90	21.08	14.98	21.51	14.98	18.42	0.037	−1.078	−0.703	−0.423	−0.300
Control group	14.54	22.70	16.31	18.77	15.60	19.37	15.60	18.74	15.25	18.72					
**Diarrhea**															
Intervention group	12.79	18.87	11.11	16.49	12.12	18.72	14.48	20.84	13.13	20.66	1.234	2.226^*^	1.342	0.843	0.256
Control group	14.96	17.53	9.83	15.30	11.11	15.81	10.26	15.48	13.67	17.35					
**Financial problems**															
Intervention group	28.62	23.33	24.58	26.34	20.88	22.12	20.88	23.13	18.18	21.44	1.621	0.905	2.485^*^	4.334^***^	5.300^***^
Control group	30.77	32.58	31.20	30.55	29.06	30.08	22.65	26.59	22.22	25.58					
**Global health**															
Intervention group	69.87	18.77	65.32	19.07	69.44	20.06	70.71	18.87	71.21	19.64	0.672	3.106^**^	1.455	0.955	0.266
Control group	69.12	21.99	61.43	23.31	62.93	26.06	64.21	19.93	66.56	28.98					

The overall F score for the interaction effect is after controlling for age, sex, BMI, and education as covariates.

The t-values indicate between-group differences at T2, T3, T4, and T5, while controlling for the T1 score.

DT, distress thermometer; QLQ-C30, Quality-of-Life Questionnaire-Core 30.

Higher scores indicate better functioning (scaled from 0–100); lower scores indicate fewer symptoms (scaled from 0–100).

Baseline (T1), 1 month (T2), 3 months (T3), 6 months (T4), and 12 months (T5) after initiation of the intervention; mean (M), standard deviation (SD).

Intervention group (IG), control group (CG).

^*^P < 0.05.

^**^P < 0.01.

^***^P < 0.001.

**Figure 3:**
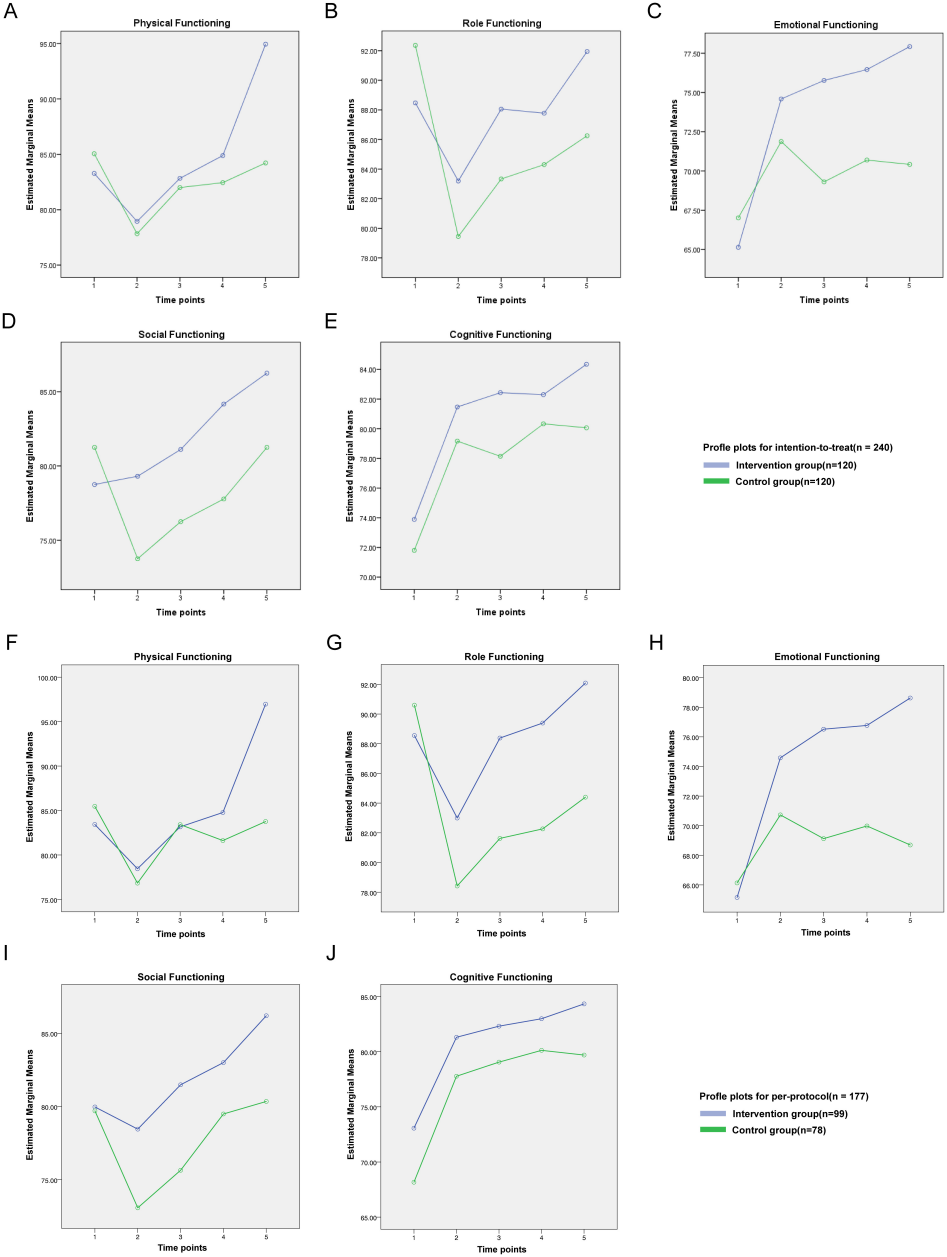
QLQ-C30 intent-to-treat outcome functional domain line chart.

Significant time-by-group interactions were observed for physical function (ITT: *F* = 5.632, *P* < 0.001, Figure 3A; PP: *F* = 6.084, *P* < 0.001, Figure 3F), role function (ITT: *F* = 3.325, *P* < 0.05, Figure 3B; PP: *F* = 2.675, *P* < 0.05, Figure 3G), emotional function (ITT: *F* = 5.543, *P* < 0.001, Figure 3C; PP: *F* = 5.566, *P* < 0.001, Figure 3H), and social function (ITT: *F* = 3.080, *P* < 0.05, Figure 3D; PP: *F* = 1.042, *P* = 0.385, Figure 3I). Cognitive function showed no interaction in either dataset (ITT: *F* = 0.419, *P* = 0.795, Figure 3E; PP: *F* = 0.167, *P* = 0.955, Figure 3J).

**Figure 4:**
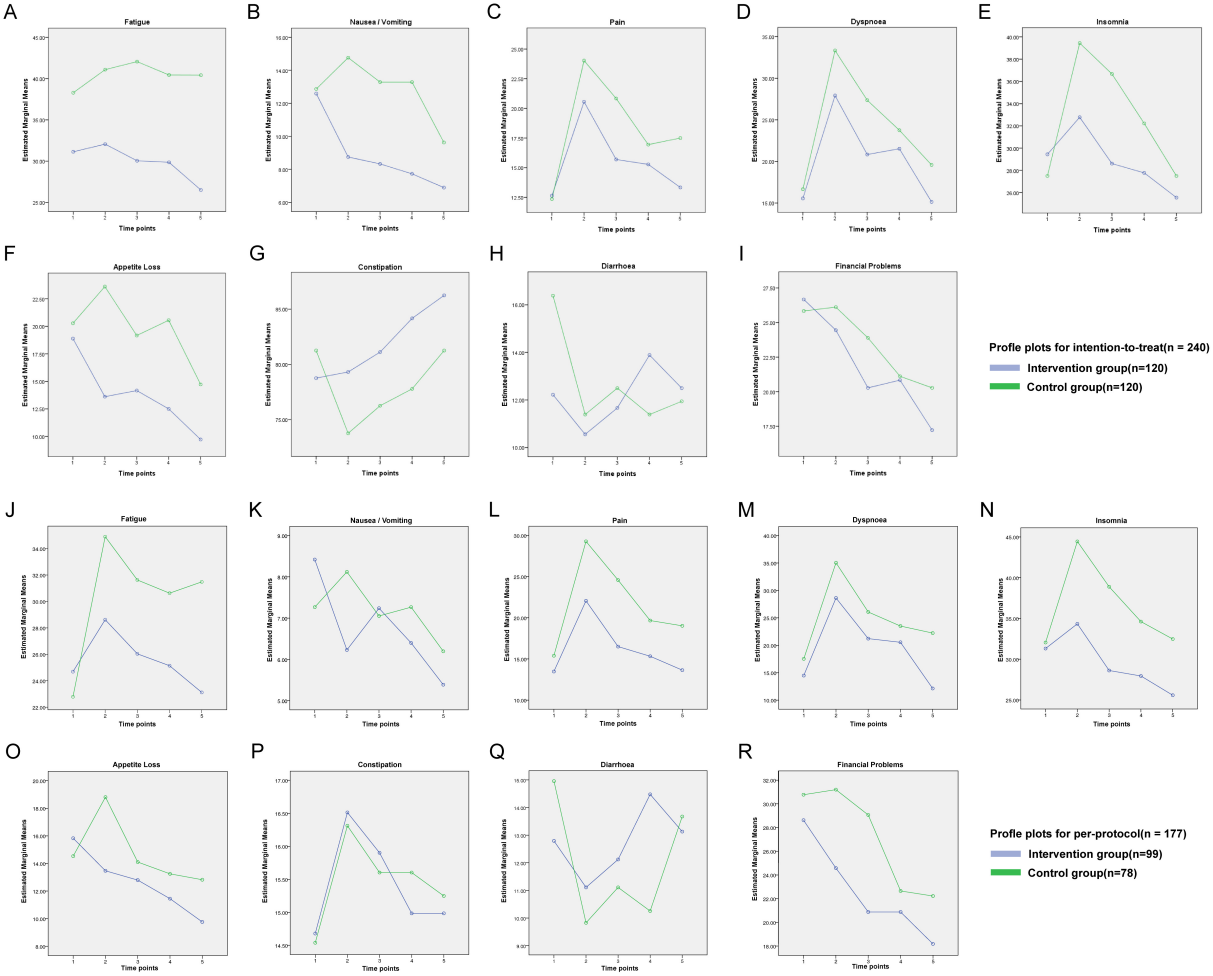
QLQ-C30 intent-to-treat outcome symptom domain line chart.

**Figure 5:**
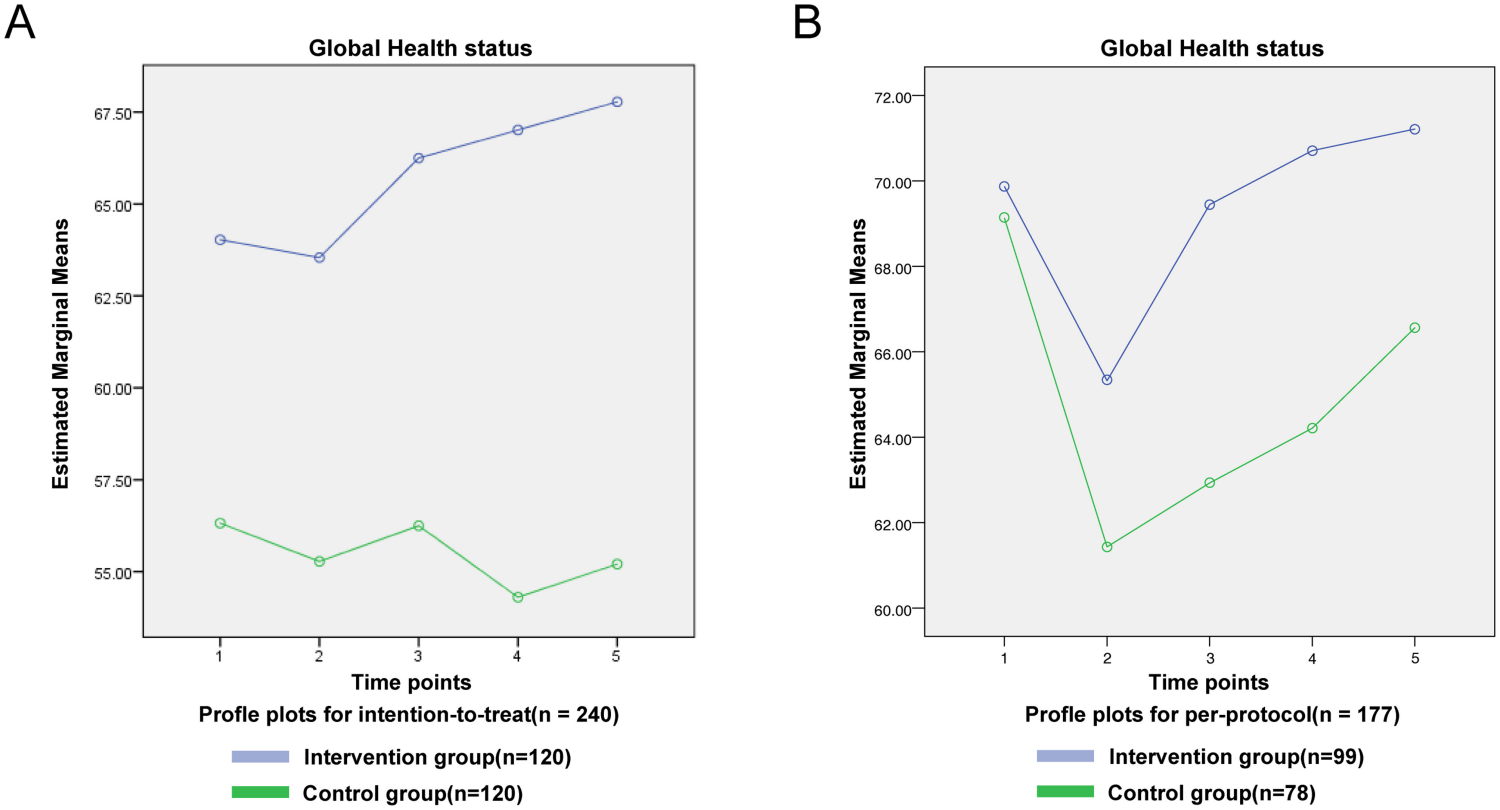
QLQ-C30 intent-to-treat outcomes global health status line chart.

For symptom scales, no significant time-by-group interactions were observed for nausea/vomiting, pain, dyspnoea, insomnia, appetite loss, constipation, diarrhea or financial difficulties in either the ITT or PP analyses (*P* > 0.05; Figures 4B–R). For fatigue, the interaction was non-significant in the ITT cohort (*F* = 1.286, *P* = 0.274; Figure 4A) but reached significance in the PP cohort (*F* = 3.464, *P* < 0.01; Figure 4J). Global health status showed no significant interaction in either dataset (ITT: *F* = 0.630, *P* = 0.642; PP: *F* = 0.672, *P* = 0.612; Figure 5).

## 4 Discussion

### 4.1 Effects of systematic intervention on patients' DT

The statistical analysis revealed a progressive decline in mean DT scores among intervention group participants from baseline to 6 months post-surgery. Notably, at the 6-month follow-up, the intervention group exhibited significantly lower DT scores compared to the control group. Both per-protocol and intention-to-treat analyses demonstrated significant time-by-group interaction effects for DT (ITT: *F* = 3.791 *P* < 0.01 PP: *F* = 5.258, *P* < 0.001), suggesting a substantial intervention effect. The PP analysis excluded 63 individuals with incomplete adherence or missing data, thereby introducing potential “healthy-adherer” bias and likely overestimating the true effect (Ahn and Kang, 2023; Giovanazzi et al., 2022). Consequently, the ITT findings remain the primary basis for inference, and the PP results are presented solely as a sensitivity analysis.

Chronic stress poses a pervasive challenge in cancer survivorship, driven by persistent fears of recurrence and treatment-related morbidity. This stress not only impairs quality of life but also predicts poorer clinical outcomes, including reduced treatment adherence and accelerated disease progression (Roberts and Karatsoreos, 2021). Addressing this burden requires interventions that simultaneously target maladaptive cognitive processes and their downstream physiological effects—a core tenet of cognitive-behavioral therapy (CBT) (Curtiss et al., 2021). Our systematic intervention programme operationalised this principle through a multi-component CBT framework designed to: (1) reduce stress reactivity via mindfulness-based techniques, (2) diminish psychological distress through cognitive restructuring, (3) enhance self-management skills via behavioral activation, and (4) promote healthful behaviors through interpersonal support mechanisms.

Participants reported significant improvements in wellbeing and interpersonal closeness, particularly following mindfulness practice and physical embrace exercises. These experiential components appeared to facilitate open family discussions about emotional struggles, suggesting that the programme's efficacy stems not only from individual cognitive-behavioral change but also from strengthened social support networks. This dual mechanism—cognitive modification coupled with relational reinforcement—highlights the programme's potential for sustainable stress reduction in cancer survivorship.

### 4.2 Effects of systematic intervention on patients' QLQ-C30

We observed significant time-by-group interactions for physical, role, and emotional functioning in both the ITT and PP analyses, indicating that the intervention conferred progressively greater improvements in these domains than usual care. With the exception of fatigue in the PP cohort, none of the symptom scales nor global health status exhibited a significant interaction.

This null finding likely reflects the cohort's early-stage profile: 97% of participants had TNM stage 0–I disease, and most were asymptomatic at baseline, having been diagnosed incidentally via low-dose CT screening. Consequently, ceiling effects in baseline scores and the absence of clinically relevant symptoms (e.g. chest pain, dyspnoea, fatigue) constrained the intervention's measurable impact on this domain. These results mirror those of Akezaki et al. (2021), who similarly observed minimal post-thoracoscopic symptomatology in early-stage patients.

ITT analyses showed that physical function declined 1 month post-surgery in both groups (intervention: 78.61 ± 15.09; control: 77.28 ± 16.64), consistent with postoperative recovery trajectories reported by Linwan et al. (2023). Scores subsequently improved from 3 months onward, converging by 12 months. However, the intervention group demonstrated significantly higher physical function scores at 12 months (87.61 ± 18.05 vs. control 80.33 ± 23.28; *P* < 0.05), aligning with Wang et al.'s (2023) findings on cognitive-behavioral therapy benefits in post-surgical lung cancer patients.

The isolated fatigue finding in PP participants (*F* = 3.464, *P* < 0.01) may reflect higher baseline fatigue and greater capacity for improvement among adherent individuals. However, the lack of corresponding effects in the ITT analysis limits its clinical generalisability and underscores the need for cautious interpretation. Taken together, these data indicate that the intervention delivers clinically meaningful gains in key functional domains without attenuating symptom burden, thereby supporting its role as a safe adjunct to standard oncological care.

A participant in the intervention group described how mindfulness practice enabled her to disengage from dysfunctional thought patterns, engage with present-moment emotions and bodily sensations, and re-evaluate her relationships with self, family and others. Such experiential insights complement the programme's structured components-health education, rehabilitation training, daily-activity guidance and psychological support-which collectively enhance illness understanding and self-efficacy.

Our findings align with a review and network meta-analysis of 186 RCTs, which suggests that multicomponent prehabilitation-integrating exercise, nutritional and psychosocial elements-may meaningfully improve health-related quality of life and reduce psychological morbidity in adults awaiting major surgery. Strengthening clinician-patient communication and providing individualized holistic care are equally essential (Shivappa et al., 2024). Throughout the 12-month observation period, one participant (0.83%) in the intervention arm died, and withdrawal rates were markedly lower in the intervention group than in the control group [20 (16.66%) vs. 42 (35%)]. This differential attrition indicates that the programme was well tolerated within routine clinical workflows. Notably, all fidelity procedures-weekly nurse-led telephone reviews and daily SMS prompts-were implemented by existing ward staff employing standard mobile platforms, entailing negligible additional resource expenditure and supporting the intervention's scalability to resource-constrained or non-specialist settings. These observations support the potential clinical applicability of our SABC-based intervention beyond the psychological domain and its suitability for integration into routine peri-operative care pathways.

### 4.3 Strengths and limitations

This study benefits from a large sample size, an extended intervention and follow-up period, and robust methodological rigor. The SABC intervention is straightforward, low-cost and easily integrated into routine care, targeting situational, emotional, physical and cognitive stress responses. By foregrounding self-management supported by family involvement and promoting sustained health-behavior change, the programme empowers both patients and their caregivers. Several participants reported that intervention practices had become habitual components of their long-term healthy lifestyles, suggesting potential for sustained behavioral outcomes.

However, generalisability is constrained by the predominance of early-stage (TNM 0–I) lung-cancer cases, limiting applicability to advanced-stage populations. Although the multi-component design incorporated family touch interventions, mindfulness and body-scan exercises, the relatively low intervention dose may have attenuated effect sizes. Future research should explore higher-intensity protocols and include more heterogeneous disease stages to clarify the intervention's full therapeutic potential.

## 5 Conclusion

The SABC intervention targets four core domains—stress-regulation capacity, amygdala reactivity, maladaptive cognition, and physical health—to induce durable neural and behavioral change. By reconfiguring stress-responsive circuits, the programme reduces negative affect, somatic symptoms, and cognitive distortions while enhancing functional competence. These mechanisms collectively mitigate psychological distress and improve physical, role, emotional, and social functioning.

The SABC intervention did not yield statistically significant improvements over usual care in the domains of cognitive function, pain, dyspnoea, constipation, financial hardship or global health status. These null effects are plausibly attributable to the composition of the study cohort: 97% of participants had stage 0–I disease and remained largely asymptomatic at baseline, thereby engendering a ceiling effect that constrained measurable change. Collectively, our findings indicate that the SABC intervention constitutes a low-cost and highly feasible adjunct to standard supportive care following lung-cancer surgery, and can therefore be adopted directly by thoracic surgical, oncology and community nursing teams.

### 5.1 Intervention fidelity and implementation

- Adherence: during the study period, one patient (0.83%) in the intervention group died, and 20 (16.66%) in the intervention group vs. 42 (35%) in the control group withdrew.
- Dose delivered: 100% of planned modules were provided to all participants.
- Dose received: daily practice logs were completed on 82.4% of possible days.
- Competence: intervention fidelity was assured through monthly expert supervision. One consultant liaison-psychiatrist and one clinical psychologist-both external to the trial team-independently reviewed 20% of audio-recorded nurse sessions against a 15-item checklist (covering content accuracy, motivational-interviewing spirit and time allocation). Each item was rated 0–2; sessions were deemed adherent only if ≥12 of 15 items scored 2. All reviewed sessions met this criterion (mean 92%, range 87%−100%).
- Contamination: no participant in the control arm accessed SABC materials during follow-up (verified by cross-checking WeChat logs and telephone records).
- Safety: no adverse events or study-related harms were observed or reported throughout the intervention and follow-up periods.

## Data Availability

The raw data supporting the conclusions of this article will be made available by the authors, without undue reservation.
